# Analysis of high pI *α*-*Amy*-*1* gene family members expressed in late maturity α-amylase in wheat (*Triticum aestivum* L.)

**DOI:** 10.1007/s11032-013-9968-z

**Published:** 2013-10-17

**Authors:** Cong-Rong Cheng, Klaus Oldach, Kolumbina Mrva, Daryl Mares

**Affiliations:** 1 School of Agriculture Food and Wine, University of Adelaide, Waite Campus, Glen Osmond, SA 5064 Australia; 2 Crop Improvement, Plant Genomics Centre, South Australian Research and Development Institute, Waite Campus, Urrbrae, SA 6064 Australia

**Keywords:** Late maturity α-amylase (LMA), High pI α-amylase, *α*-*Amy*-*1* genes, *Triticum aestivum*, Wheat, Quantitative real-time RT-PCR

## Abstract

Late maturity α-amylase (LMA) is a genetic defect involving the synthesis of high pI isozymes of α-amylase encoded by *α*-*Amy*-*1* genes during the later stages of grain development. The aims of this investigation were to determine both the number of expressed *α*-*Amy*-*1* genes and their relative transcript abundance. Sub-cloning and sequencing of expressed high pI α-amylase genes in developing wheat seeds revealed three insertion/deletion patterns in the 3′ untranslated region and numerous single nucleotide polymorphisms at the 3′ end of *α*-*Amy*-*1.* The genetic variations defined 36 *α*-*Amy*-*1* gene sequences that were expressed on the onset of LMA in doubled haploid progenies (SpM25, SpM52 and SpM127) derived from the cross Spica (LMA)/Maringa (non-LMA). Five isoelectric point groups were predicted based on the translated partial coding sequences. The potential application of quantitative real-time RT-PCR in screening wheat genotypes for LMA is discussed.

**Electronic supplementary material:**

The online version of this article (doi:10.1007/s11032-013-9968-z) contains supplementary material, which is available to authorized users.

## Introduction

Alpha-amylase (EC number 3.2.1.1: 1,4-α-d-glucan glucanohydrolase), an endo-hydrolase which belongs to glycoside hydrolase family 13, acts on α-1,4-glycoside linkages of starch (Davies and Henrissat 1995). In cereals, α-amylase is most commonly associated with the germination of the grain. During germination, gibberellin is synthesized in the embryo, travels to the scutellum and into the aleurone layer. The aleurone layer surrounding the endosperm is triggered to synthesize a range of hydrolytic enzymes including α-amylase to mobilize starch and other stored reserves. Starch is broken down into simple sugars and transported back to the embryo for the use of the growing seedling (Ritchie et al. 2000).

The major α-amylases in wheat (*Triticum aestivum* L.) are the high and low isoelectric point (pI) α-amylases, encoded by the *α*-*Amy*-*1* and *α*-*Amy*-*2* genes, respectively. They have previously been referred to as germination and developmental or green isozyme, respectively, due to the appearance of the former during the germination stage and the latter during early grain development (Gale and Ainsworth 1984). The *α*-*Amy*-*1* genes have been mapped to chromosomes 6A, 6B and 6D using the Chinese Spring nullisomic-tetrasomic lines and based on isoelectric focusing of α-amylase isozymes produced following treatment of grains with gibberellic acid (Gale et al. 1983). The *α*-*Amy*-*2* genes, on the other hand, have been assigned to chromosomes 7A, 7B and 7D using the same method (Gale et al. 1983; Nishikawa et al. 1988). Other methods involving Southern Blot analysis have yielded similar results (Lazarus et al. 1985). In another similar experiment, up to 27 allelic variations of *α*-*Amy*-*1* and *α*-*Amy*-*2* genes have been proposed (Ainsworth et al. 1985). However, not all isozymes could be assigned to a particular chromosome using these methods and the genetic control of these isozymes remains unclear. Due to an inconsistent nomenclature of high and low pI α-amylase gene products in wheat and barley literature, we will herein refer to high pI or low pI α-amylase isozymes rather than AMY-1 and AMY-2.

Late maturity α-amylase (LMA) is a genetic defect involving the synthesis of high pI α-amylase during the middle to later stages of wheat grain development in the absence of germination, resulting in mature grain with high pI α-amylase and low falling number (Mares and Mrva 2008). LMA can occur under normal conditions, or be induced by cool temperature shock during the middle stages of grain development (Mrva et al. 2006; Mrva and Mares 2001a). Grains with low falling number may cause processing and storage problems as well as having adverse effects on the quality of the end-products. Grains with low falling number will be downgraded to feed grade, causing economic losses to growers (Mares and Mrva 2008; Edwards et al. 1989). Although present in only a small number of Australian commercial wheat varieties, the frequency of wheat cultivars with LMA phenotype is high in breeding programs both in Australia and overseas and requires careful monitoring and management. The detection and measurement of α-amylase in grains has evolved over the years from the falling number method, to assays involving dye-labelled starch, to isoelectric focusing, and to the development of monoclonal and polyclonal antibodies, which are currently used in an enzyme-linked immunosorbent assay (ELISA) format (Mares and Mrva 2008; Hagberg 1960, 1961). A method of detection of high pI α-amylase by quantitative RT-PCR has not been developed mainly due to the limited information on the gene sequences coding for high pI α-amylase isozymes in wheat.

Analysis of a Cranbrook (LMA)/Halberd (non-LMA) doubled haploid population revealed two LMA quantitative trait loci (QTL) on chromosomes 3B and 7B (Mrva and Mares 2001b; Mrva et al. 2009). These studies represent the first steps towards development of molecular tools for marker-assisted selection against LMA genotypes and the elimination of LMA from breeding programs (McNeil et al. 2009). However, studies that contribute towards the understanding of the mechanisms involved in LMA in wheat are still lacking. Recent research has reported some quite dramatic physiological, transcriptomic and hormonal changes that occur during LMA (Barrero et al. 2013). Additionally, information on the specific wheat high pI α-amylases expressed in LMA is also lacking. Even for germination and GA-challenged grain or aleurone, there is currently limited information on the genomic DNA, cDNA and protein sequences of the wheat high pI α-amylase isozymes in publicly available databases. Furthermore, the total number of high pI α-amylase isozymes present in wheat and the number of gene copies are not known.

In parallel with the study reported by Barrero et al. (2013), this study aimed to identify and analyze the number of expressed *α*-*Amy*-*1* genes in LMA-prone bread wheat lines selected from a doubled haploid population, Spica (tall, LMA)/Maringa (Rht1 isoline, non-LMA), with Spica as female parent. The possibility of using RT-PCR to detect the specific expression of high pI α-amylase genes was also investigated.

## Materials and methods

### Plant material

Sets of tall genotypes with and without LMA were selected from a doubled haploid population, Spica *rht* (LMA)/Maringa *Rht1* (non-LMA), based on consistent phenotype over several seasons and alleles at the LMA 7B QTL (Mrva and Mares 2001b). Doubled haploid lines SpM47, SpM84 and SpM109, which have a non-LMA phenotype, were compared with lines SpM25, SpM52 and SpM127, which express constitutive LMA. Tall genotypes were selected to avoid the confounding effects of the semi-dwarfing, GA-insensitive gene *Rht1* on LMA expression (Mares and Mrva 2008). Plants were grown side by side in pots in a glasshouse and spikes tagged at anthesis. Spikes (10 per sampling time) were sampled at 12, 17, 20, 23, 26, 29, 32, 35, 40 and 45 days post-anthesis (dpa) for determination of grain moisture, grain dry weight, grain appearance, high pI α-amylase abundance and isolation of aleurone tissue for preparation of mRNA. Between anthesis and grain maturity, the mean minimum and maximum temperatures were 16 °C (range 12.8–20.5 °C) and 25.3 °C (range 21.4–31.4 °C) respectively.

### Determination of grain moisture and dry weight

Duplicate samples of 20 grains were removed from the spikes, weighed, dried at 100 °C for 2 days and weighed again. Grain moisture content was expressed as percent fresh weight, calculated from the difference between fresh and dried weights (Barrero et al. 2013).

### Extraction and ELISA determination of high pI α-amylase protein

Eight replicates each of five de-embyronated grains per sampling time were crushed, mixed with 1 mL 0.85 M NaCl containing 0.018 M CaCl_2_ on a vortex mixer, incubated at 37 °C overnight, then centrifuged for 10 min at 14,000 rpm in a microcentrifuge. An aliquot of 100 μL was used in the microplate ELISA (Barrero et al. 2013).

High pI α-amylase protein was assayed in a 96-well plate format using a modification of the sandwich ELISA reported by Verity et al. (1999). Plates were coated with a rabbit anti-wheat α-amylase polyclonal antibody, blocked with bovine serum albumin, incubated with extracts of grain, washed, and then incubated with a mouse anti-barley high pI α-amylase monoclonal antibody. The plates were again washed and incubated with horse radish peroxidase (HRP)-labeled donkey anti-mouse antibody (Sigma) before adding colour developer, TMB (3,3,5,5′-tetramethylbenzidine) substrate (Elisa Systems Pty Ltd, QLD, Australia), then recording optical density (OD) at 595 nm in a microplate reader. High pI α-amylase protein content of grains was expressed as the mean OD of the eight microplate wells corrected for background with grain from a bulk sample of Sunco, a non-LMA control cultivar.

The polyclonal and monoclonal antibodies are currently maintained by the South Australian Research & Development Institute, South Australia and made available under a research-only agreement between the Grains Research & Development Corporation of Australia and Bayer AG, the holder of the patent covering the use of these antibodies for determination of α-amylase in wheat (Barrero et al. 2013).

### Preparation of mRNA and cDNA

Grain aleurone including the grain coat was prepared over dry ice from de-embryonated grains according to the method described by Mrva et al. (2006) and immediately frozen in liquid nitrogen. mRNA and cDNA were prepared by Drs J. Barrero and F. Gubler at CSIRO Plant Industry, Canberra, as part of a joint parallel investigation of molecular and physiological aspects of LMA. mRNA was prepared using the hexadecyltrimethylammonium method described by Chang et al. (1993) and used to synthesise cDNA using SuperScript III (Invitrogen Life Sciences) as described in Barrero et al. (2013).

Seeds from line SpM47 (non-LMA) and line SpM52 (LMA) were used for genomic sequence analysis of *α*-*Amy*-*1*. For DNA extraction, seeds were germinated and grown for 5 days, and three leaf segments approximately 4 cm long were harvested from both lines. Leaf samples were snap-frozen in liquid nitrogen and DNA extracted as previously described (Williams et al. 2006).

### Bioinformatics

Barley (*Hordeum vulgare*) high pI α-amylase genomic DNA (gi:166984, 166994) and protein sequences (gi:166985, 166995) were obtained from the National Center for Biotechnology Information (NCBI http://www.ncbi.nlm.nih.gov/) and Expert Protein Analysis System proteomics server (ExPASy http://expasy.org/) online database. These sequences were used as queries to screen the wheat Expressed Sequence Tags (EST) database using the Basic Local Alignment Search Tool (BLAST) (Altschul et al. 1997). Best-matching wheat ESTs with an *E*-value lower than 1E-70 were assembled using the software Contig-Express in Vector NTI Suite (Invitrogen, Carlsbad, CA, USA) to obtain a predicted wheat α-amylase sequence. The online tool ClustalW2 (http://www.ebi.ac.uk/Tools/msa/clustalw2/) was used for multiple sequence alignment (Larkin et al. 2007). The phylogenetic tree was constructed using the neighbour-joining method and default tree format using the same online tool. Translation of nucleotide sequences and prediction of isoelectric points were carried out using Vector NTI. The phylogenetic tree was generated using online program TreeDyn 198.3 (Dereeper et al. 2010).

### Primer design, sub-cloning and sequencing

Primers HpI1_F and HpI1_R; HpI2_F and HpI2_R; HpI3_F and HpI3_R were designed to amplify the predicted *α*-*Amy*-*1* gene sequence as three overlapping fragments using PCR (Table S1). Two separate 10 μL PCR reactions were carried out, using 0.25 units *Taq* DNA polymerase (Qiagen, VIC, Australia), 1× Taq buffer, 1.5 mM MgCl_2_, 0.2 mM of dNTP and 0.2 μM of each primer (denaturing step at 95 °C for 2 min, 38 cycles of 94 °C for 30 s, 58 °C for 30 s and 72 °C for 1 min, and final step at 72 °C for 5 min). Fragments were sub-cloned into a cloning vector (TOPO pCR8) according to the manufacturer’s protocol (Invitrogen). The plasmids were transformed into chemically competent *E. coli* DH5α cells, plated on selective agar plates [50 mL Luria–Bertani medium, 50 μL IPTG (isopropyl thiogalactosidase), 50 μL ampicillin, 100 μL X-gal (5-bromo-4-chloro-3-indolyl-β-d-galactopyranoside) per plate] and incubated at 37 °C for 16–20 h. One hundred white bacterial colonies were picked and used as templates for PCR using the standard vector primers M13F and M13R. PCR amplicons were purified using Millipore PCR cleanup filter plates (Millipore, NSW, Australia) prior to sequencing using the nested T3 and T7 primers and BigDye3.1 reaction mix (Applied Biosystems, CA, USA). Sequencing reaction products were separated on an ABI3730 capillary sequencer by the Australian Genome Research Facility (AGRF). For further confirmation, PCR on genomic DNA with primers HpI3_F and HpI3_R was repeated with a proof-reading DNA polymerase. A total of three separate 12.5 μL PCR reactions were carried out, using 0.5 units Phusion^®^ high-fidelity DNA polymerase (New England Biolabs, QLD, Australia), 1× Phusion GC Buffer, 0.25 μM of each primer, 0.2 mM dNTPs and 3 % DMSO (denaturing step at 98 °C for 30 s, 30 cycles of 98 °C for 10 s, 62 °C for 30 s and 72 °C for 30 s, and final step at 72 °C for 10 min). PCR products were subjected to A-tailing prior to subcloning using 0.25 units *Taq* DNA polymerase, 1× Taq buffer, 1.5 mM MgCl_2_ and 0.2 mM of dATP (72 °C for 30 min).

### Genetic analysis

Nullisomic-tetrasomic Chinese Spring wheat lines n6A-t6B, n6A-t6D, n6B-t6A, n6B-t6D, n6D-t6A and n6D-t6B were used in this study (Sears 1964) to map individual *α*-*Amy*-*1* gene family members. Primers HpI_Grp1_R, HpI_Grp2_R and HpI_Grp3_R were used as reverse primers in combination with the common forward primer HpI3_F (Table S1).

### Quantitative real-time RT-PCR

Primers HpI3_F and HpI3_R that amplify all three fragments of 408, 423 and 462 bp from cDNA were used to assess the expression profile of the high pI α-amylase sequences in LMA and non-LMA wheat lines. cDNA prepared at four sampling times from LMA lines (SpM25, SpM52 and SpM127), spanning the observed onset of synthesis of high pI α-amylase protein, and non-LMA lines (SpM47, SpM84 and SpM109) were used as templates for RT-PCR analysis. RT-PCR reactions were carried out using iQ™ SYBR^®^ Green Supermix (Bio-Rad, CA, USA) according to the manufacturer’s instructions. To normalize the raw data of primers Hpi3_F/_R, cDNAs were normalized using primers Ta2291_F and Ta2291_R amplifying Ta2291 (ADP-ribosylation factor) as a recommended reference gene in wheat (Paolacci et al. 2009). Three biological replicates of the six plant lines with four time points each (17, 20, 23 and 26 dpa) were carried out with three technical repeats each.

### Sequencing

Primers HpI3_F and HpI3_R were used in end-point PCR reactions on cDNA template from pooled time points with high synthesis of high pI α-amylase (SpM25, SpM52 and SpM127 all at 23 dpa and 26 dpa) and the wheat line with the highest synthesis of high pI α-amylase (SpM52 at 23 dpa). The amplified fragments were sub-cloned into cloning vector TOPO pCR8 and transformed into *E. coli* for selection as described above. Twenty-four colonies with inserts from cDNA of LMA lines SpM25, SpM52 and SpM127 each and 95 white bacterial colonies from SpM52 were picked and served as templates using the standard vector primers M13F and M13R and subsequent sequencing reactions with nested T3 and T7 as described above. Sequences were analyzed using Contig-Express and AlignX applications of the Vector NTI software. Isoelectric points of the translated partial sequences, using the standard genetic code, were predicted with Vector NTI.

## Results

### High pI α-amylase protein synthesis during grain development

High pI α-amylase protein was detected in the grain of LMA lines beginning at 18–21 dpa in 2008 and at 20–23 dpa in 2009. ELISA OD increased rapidly to a maximum of 0.4–0.45 OD units around 27–30 dpa in 2008 and to 0.5–0.7 OD units by 29–32 dpa in 2009, and then remained constant within experimental error until harvest ripeness, with 12 % grain moisture at 45 dpa in both experiments. At 21 dpa the mean moisture content of the 2008 samples was 56 % and the grain had reached 75 % of maximum dry weight. In 2009, the mean moisture content and percent of max dry weight at 20 dpa were 62 and 60 %, respectively. No high pI α-amylase protein was detected at any stage in the non-LMA lines but the time courses of change in grain moisture and grain dry weight were very similar to the LMA lines (Barrero et al. 2013).

In this study, SpM52 and SpM47, which showed consistent LMA and non-LMA phenotypes, respectively, were used for high pI α-amylase genomic DNA analysis. Samples from non-LMA lines (SpM47, SpM84 and SpM109) and LMA lines (SpM25, SpM52 and SpM127) from the 2009 experiment were selected for high pI α-amylase gene expression analysis using quantitative real-time RT-PCR.

### Analysis of *α*-*Amy*-*1* sequences and expressed high pI α-amylase

**Fig. 1:**
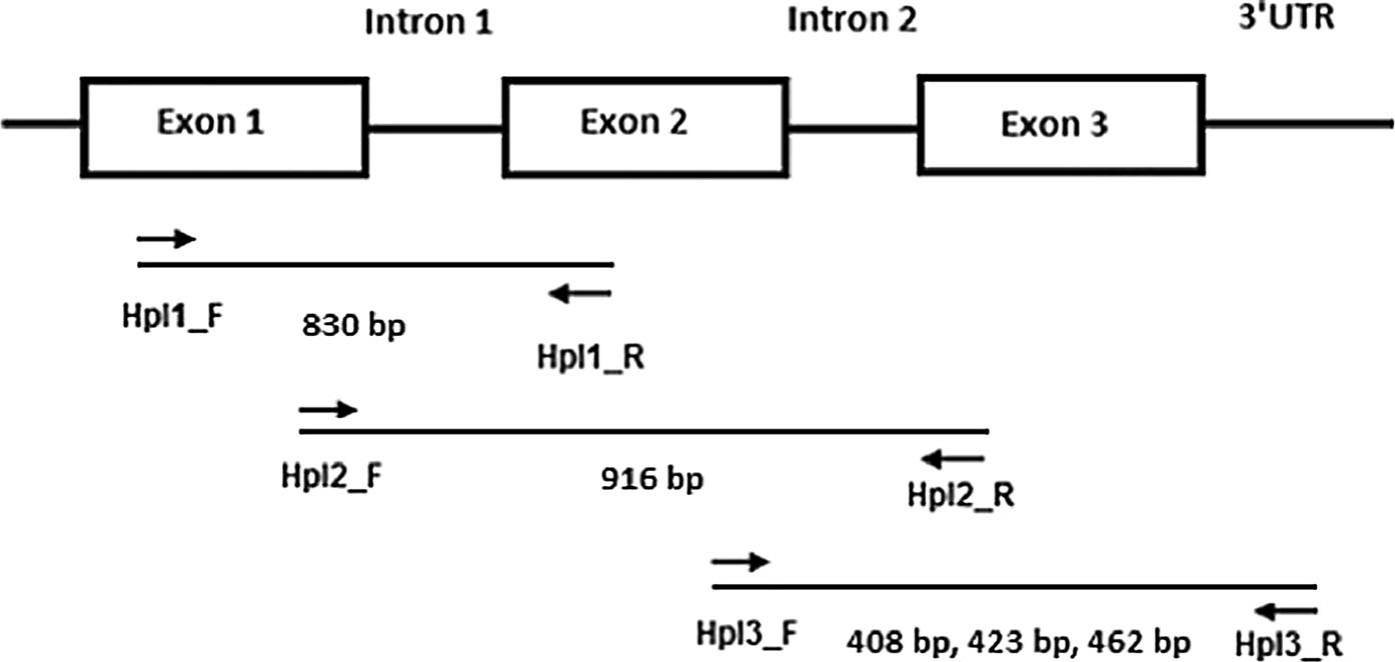
Schematic diagram of the genomic structure of *α*-*Amy*-*1* predicted by sequence comparisons with a barley high pI α-amylase gene (gi:166984). The position and orientation of the primers are indicated by *arrows*

**Fig. 2:**
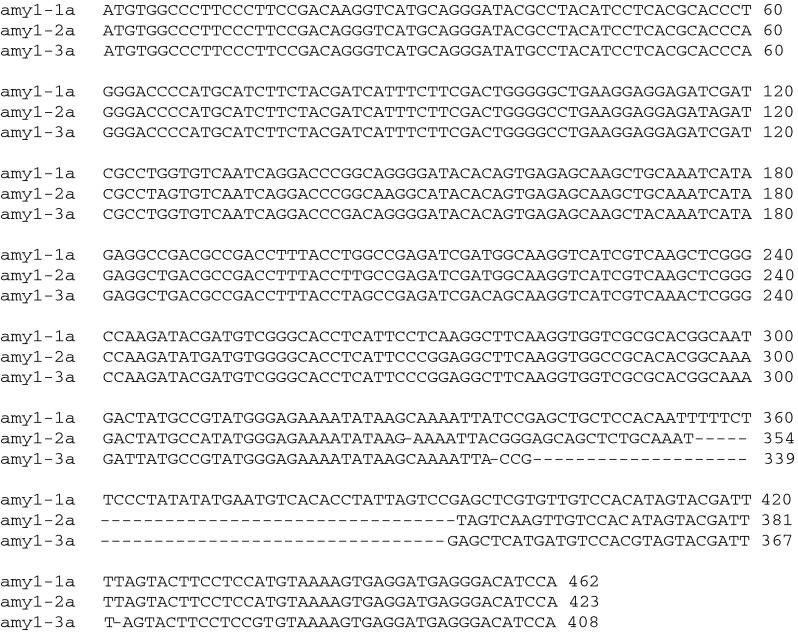
Multiple sequence alignment of representative gene family members *amy1*-*1a*, *amy1*-*2a* and *amy1*-*3a*

Barley high pI α-amylase sequences were used as queries to collate matching wheat ESTs and predict an *α*-*Amy*-*1* gene sequence. Due to the relatively large estimated gene sequence, primers were designed to amplify three overlapping fragments from Spica/Maringa DH lines SpM47 and SpM52 (Fig. 1). The amplified and subcloned genomic products were sequenced to assemble nearly full-length *α*-*Amy*-*1* sequences. Analysis of the sequenced genomic fragments revealed four single nucleotide polymorphisms (SNPs), indicating a highly conserved region in the 5′ half of the gene, whereas SNPs were frequently detected towards the 3′ end of the sequence. Furthermore, there were three patterns of insertions/deletions (InDels) in the 3′ untranslated region (3′UTR), producing different length PCR fragments when using primer pair HpI3_F/R (Fig. 2).

Analysing these 3′ sequences using cDNA from samples collected during LMA expression yielded 36 different *α*-*Amy*-*1* gene sequences from the three LMA wheat lines. The 36 different sequences were derived from an initial 167 cDNA sequences representing 24 clones each of lines SpM25, SpM52 and SpM127, and an additional 95 clones of line SpM52. Due to the three InDel patterns in the 3′UTR (Fig. 2), fragments with sizes 408, 423 and 462 bp were obtained and analyzed. Clones with sub-optimal quality and clones appearing only once were discarded from further analysis to avoid overestimation of the *α*-*Amy*-*1* sequence diversity as a consequence of poor quality or limited *Taq*-polymerase fidelity, resulting in 91 high-quality sequences from SpM52. Of these, 22 sequences formed nine unique sequences and are referred to as *amy1*-*1a*—*amy1*-*1i* (462 bp), 50 sequences formed 23 unique sequences and are referred to as *amy1*-*2a*—*amy1*-*2w* (423 bp), and 19 sequences formed four unique sequences and are referred to as *amy1*-*3a*—*amy1*-*3d* (408 bp). Sequence alignment was carried out using a multiple sequence alignment online tool, ClustalW2 (Fig. S1a-c).

**Fig. 3:**
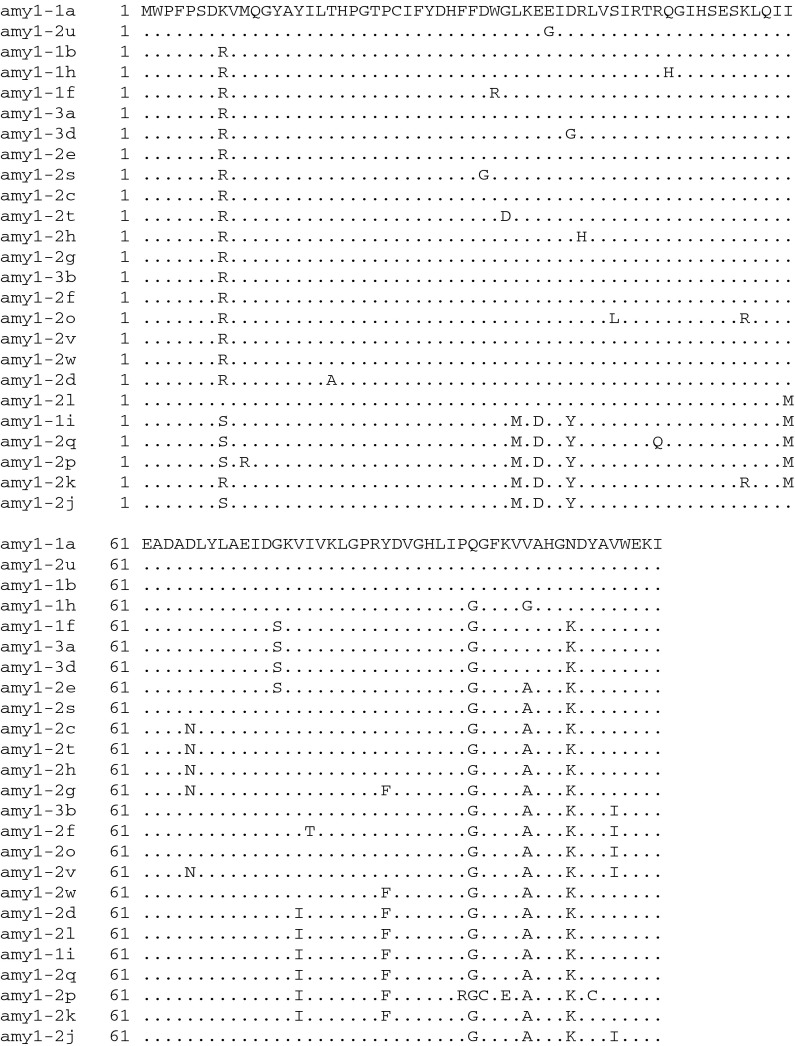
Multiple sequence alignment of the 25 unique amino acid partial sequences. Consensus nucleotides are indicated by ‘*dot*’

The translation of these 36 unique partial cDNA sequences revealed 25 unique amino acid sequences due to the degenerate nature of the genetic code leading to ‘silent’ variations (Fig. 3). The predicted isoelectric points of the partial protein sequences were indicative of five isoelectric point groups based on the assumption that the remaining protein sequence remains conserved, as suggested by the sequenced 5′ regions (Table S2).

**Fig. 4:**
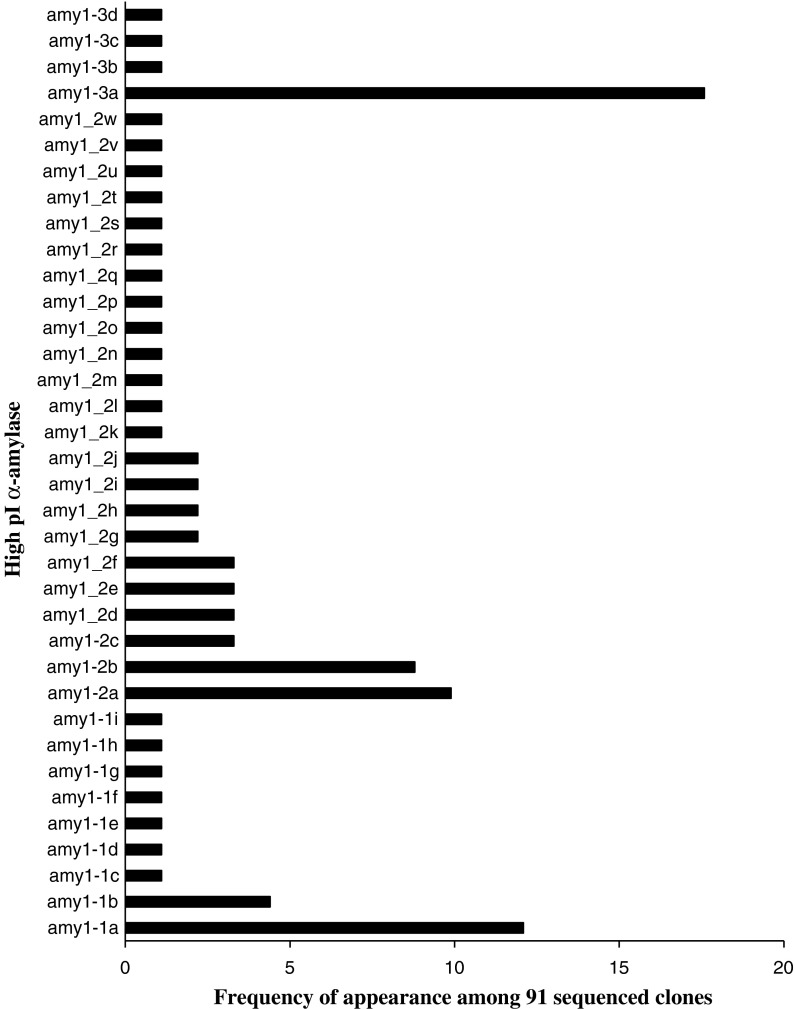
The frequency of expression of different *α*-*Amy*-*1* gene sequences during LMA based on sequencing of 91 clones in line SpM52

During LMA, the 36 gene family members of high pI α-amylase were not equally expressed. *Amy1*-*1a*, *amy1*-*1b*, *amy1*-*2a*, *amy1*-*2b* and *amy1*-*3a* were more commonly expressed in line SpM52 (Fig. 4). These sequences were submitted to Genbank and each assigned a Genbank id: KF581187, KF581188, KF581189, KF581190 and KF581191, respectively (Table S3).

### Analysis of DNA polymerase proof-read sequences

In order to address the possibility that the large number of observed sequence variants is due to the limited fidelity of *Taq* polymerase, an additional 95 high-quality sequences using primers HpI3_F/_R were analysed using a proof-reading enzyme, which has a claimed 50× higher fidelity than the standard *Taq* (Frey and Suppmann 1995). Genomic DNA from LMA lines SpM25, SpM52 and SpM127 was used as template producing 31 sequences matching six gene family members previously identified: *amy1*-*1a*, *amy1*-*2b, amy1*-*2c*, *amy1*-*2k*, amy1-*2l* and *amy1*-*2m.* A greater number of genetic variations was apparent among these clones derived from genomic templates compared to the previous analysis using cDNA (Fig. S1a, b, c). This difference is expected as genomic clones are derived from expressed and non-expressed genes and pseudogenes.

### Confirming the identity of high pI α-amylase

In a BLAST search against barley non-redundant nucleotides using the 36 unique gene family members as queries, barley high pI α-amylase gene *amy46* had a higher sequence similarity to both the *amy1*-*1* and *amy1*-*3* groups of genes whereas barley high pI α-amylase gene *amy6*-*4* had a higher sequence similarity to the *amy1*-*2* group of genes. Multiple sequence alignment and phylogenetic analysis also revealed a higher sequence similarity and genetic relationship between the wheat and barley partial DNA sequences (3′ region) for high pI α-amylase (Figs. S2 & S3).

### Genetic mapping using Chinese Spring nullisomic-tetrasomic lines

Amplification using the three primer pairs specific to the three InDel groups in the 3′UTR on nullisomic-tetrasomic Chinese Spring lines showed that the group with the largest deletion (408 bp) was associated with chromosome 6D (Fig. S4). The group containing no deletion (462 bp) was mapped to chromosome 6A (Fig. S4). No specific mapping position was obtained for the group with the length of 423 bp. The primer pairs are formed between HpI3_F (5′-ATGTGGCCCTTCCCTTCCGA-3′) and HpI_Grp1_R (5′- GTGGACATCATGAGCTCCGGTAA-3′), or HpI_Grp2_R (5′- GTGGACAACATGACTAATTTGCAGAG-3′), or HpI_Grp3_R (5′- ACAACACGAGCTCGGACTAATAGG-3′) to amplify fragments of 356, 370 and 405 bp, respectively.

### Transcript levels of *α*-*Amy*-*1* in LMA and non-LMA wheat

**Fig. 5:**
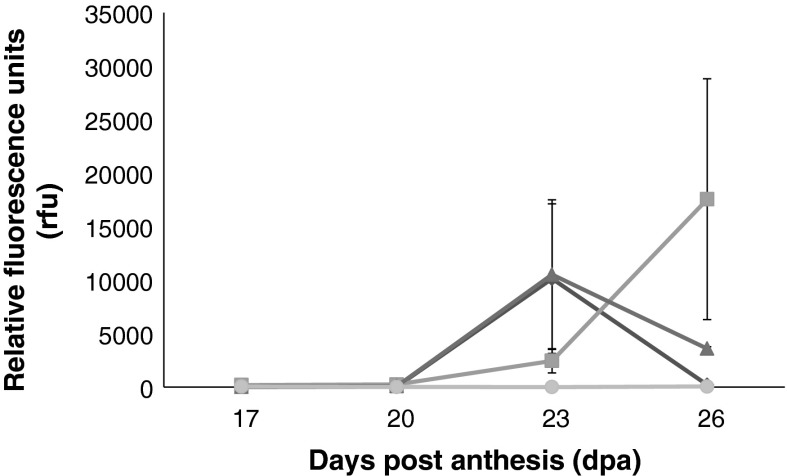
Normalized expression levels of *α*-*Amy*-*1* in LMA lines SpM25 (*filled diamond*), SpM52 (*filled square*) and SpM127 (*filled triangle*) at 17, 20, 23 and 26 dpa. Expression levels in non-LMA SpM109, SpM47 and SpM84 are represented by (*filled circle*) with values not significantly different from zero. *Error bars* denote ± standard errors

The relative abundance of *α*-*Amy*-*1* transcripts in all six cDNA samples (non-LMA lines SpM47, SpM84, SpM109 and LMA lines SpM25, SpM52, SpM127) was determined by performing RT-PCR using primers HpI3_F and HpI3_R, which were designed based on the consensus sequence from previous *α*-*Amy*-*1* genomic DNA sequencing. RT-PCR analysis showed that no significant transcript levels of *α*-*Amy*-*1* were detected in cDNAs from non-LMA lines SpM47, SpM84 and SpM109 at any of the four time points (17, 20, 23 and 26 dpa) measured, whereas all three LMA-prone lines showed a clear increase of high pI α-amylase transcript levels during the time course (Fig. 5).

## Discussion

With the limited information available on the genomic DNA, cDNA and protein sequences of wheat high pI α-amylases in the National Center for Biotechnology Information (NCBI http://www.ncbi.nlm.nih.gov/) and Expert Protein Analysis System proteomics server (ExPASy http://expasy.org/) online databases, the known genomic and protein sequences of barley (*Hordeum vulgare*) α-amylase were used to search for high pI α-amylase genes in wheat. Similar to wheat, barley α-amylases are divided into high pI and low pI α-amylases. The ability to distinguish the high pI and the low pI α-amylase is crucial as it is only the high pI α-amylase isozymes that are active in lines with the LMA quality defect. The barley high pI α-amylase sequences were used to screen for corresponding wheat ESTs. Multiple sequence alignment showed a higher degree of nucleotide similarity with barley high pI α-amylase than low pI α-amylase, confirming their identity.

Sequence analysis of the genomic clones of high pI α-amylase genes revealed that most SNPs between the subcloned gene fragments were distributed near the 3′ end region of the sequences. These SNPs defined a total of 36 gene sequences which are being expressed during LMA in progeny lines from the cross Spica (LMA)/Maringa (non-LMA). Sequences with a single SNP appearance were deleted from analysis to eliminate *Taq* fidelity issues. In addition, replacing a non-proof-reading *Taq* DNA polymerase with a proof-reading, high-fidelity DNA polymerase did not reduce the occurrence of SNPs in the sequences. Although there were only six gene family members that were confirmed with the proof-reading DNA polymerase-generated sequences, the level of sequence diversity among the clones generated with the proof-reading polymerase is also large. This is not surprising as all genomic high pI α-amylase genes including pseudogenes serve as PCR templates whereas in the cDNA analysis only expressed genes will be amplified by PCR.

Matthies et al. (2009) discovered six SNPs that defined four haplotypes in the high pI α-amylase (*α*-*Amy*-*1*) genes among 117 European spring and winter barley cultivars. Taking into account the hexaploid character of wheat, the number of variations observed in the bread wheat used in this study is disproportionally higher than if diploid barley is used to estimate the number of *α*-*Amy*-*1* genes expected in wheat.

Analysis of the partial translated protein sequences at the 3′ end revealed five different isoelectric point groups, which appears to be consistent with the observation of four major and one faint band/isozyme of high pI α-amylase in LMA lines obtained by isoelectric focusing (Mares and Gale 1990). Given the more conserved nucleotide sequence upstream of this partial DNA sequence, it is less likely for the few SNPs in the upstream region to alter the isoelectric point. The differences in the calculated isoelectric points have helped explain part of the observed isoelectric focusing (IEF) patterns obtained for wheat high pI α-amylase. Gale et al. (1983) were not able to map all high pI α-amylases to a specific sub-genome. We mapped the group with the largest deletion in the 3′UTR (*amy1*-*3*) to chromosome 6D and the group with no deletion (*amy1*-*1*) to chromosome 6A even though these two groups contain sequences with different predicted isoelectric point groups. Therefore, it appears that protein sequences with the same isoelectric point can be encoded by different sub-genomes, which explains the difficulty of mapping all isozymes with a specific isoelectric point to a particular chromosome.

In comparison to the *amy1*-*1* and *amy1*-*2* described in Barrero et al. (2013), the primers for *amy1*-*2* (designed based on ESTs CJ693813 and CA724050) bind to groups *amy1*-*1*, *amy1*-*2* and *amy1*-*3* (described in this study) with 1–3 mismatches, amplifying products of sizes 171 bp, 132 bp and 118 bp, respectively. Furthermore, both the ESTs CJ693813 and CA724050 have high sequence similarity, especially towards group *amy1*-*1* described here (data not shown). However, the reverse primer for *amy1*-*1* (Barrero et al. 2013) did not match any of our sequences.

The *α*-*Amy*-*1* expression detected in our RT-PCR study was consistent with the results from high pI α-amylase protein detection using ELISA. In both cases, the expression of high pI α-amylase was at the later stages of grain development. However, due to the short-lived nature of mRNA in comparison to the more stable proteins (Barrero et al. 2013), the peak of expression of high pI α-amylase preceded the protein expression profile and occurred only over a small window. From a practical point of view, this would require the collection of RNA samples at several time points to ensure the capture of the mRNA levels of high pI α-amylase. However, given the close correlation of the RT-PCR assay to the ELISA, it could potentially serve as an alternative method of detecting LMA-prone lines among breeding lines. Nevertheless, the primers should be improved and optimized further in a wider range of LMA genotypes to confirm their correlation with the LMA phenotype. It would also be of interest to compare the expression patterns observed in LMA affected grain with early germination.

In conclusion:

1. There are at least 36 *α*-*Amy*-*1* gene family members defined by at least 50 SNPs in Spica/Maringa that are expressed during LMA. It is possible that different alleles or abundances exist in other genotypes.
2. The 36 gene family members expressed indicate gene products with five isoelectric point groups in LMA prone genotypes.
3. The different α-*Amy*-*1* genes are not expressed equally in LMA.
4. RT-PCR appears to be an alternative method for detecting LMA phenotypes.

## Electronic supplementary material

Below is the link to the electronic supplementary material.

Supplementary material 1 (PDF 364 kb)
