# Supplementary material for: Analysis of high pI α-Amy-1 gene family members expressed in late maturity α-amylase in wheat (Triticum aestivum L.)
Source: Mol Breed. 2013 Oct 17;33(3):519–29. doi: 10.1007/s11032-013-9968-z (PMC3918125; doi:10.1007/s11032-013-9968-z)
Supplement: Supplementary file 1 — Supplementary material 1 (PDF 364 kb) [file 11032_2013_9968_MOESM1_ESM.pdf]

Table S1. Forward (F) and reverse (R) primer pair sequences used to amplify fragments of the predicted *α-Amy-1* gene sequence.

**Primer**	**Primer Sequence (5'-3')**	**Fragment size (bp)**
HpI1_F	TCGTCCTCCTTGGCCTGTCG	830 bp
HpI1_R	CGGAGCCGCCCACCTTGTTC	
HpI2_F	ATGATCTGCCGCGACGACCG	916 bp
HpI2_R	CGGCCACCTTGAAGCCTCCG	
HpI3_F	ATGTGGCCCTTCCCTTCCGA	408 bp, 423 bp and 462 bp
HpI3_R	TGGATGTCCCTCATCCTCACTTTTACA	
HpI3_F	ATGTGGCCCTTCCCTTCCGA	356 bp
HpI_Grp1_R	GTGGACATCATGAGCTCCGGTAA	
HpI3_F	ATGTGGCCCTTCCCTTCCGA	370 bp
HpI_Grp2_R	GTGGACAACATGACTAATTTGCAGAG	
HpI3_F	ATGTGGCCCTTCCCTTCCGA	405 bp
HpI_Grp3_R	ACAACACGAGCTCGGACTAATAGG	
T a2291_F	GCTCTCCAACAACATTGCCAAC	165 bp
T a2291_R	GCTTCTGCCTGTCACATACGC	

```
amy1-1a    1 GGGACCCCATGCATCTTCTACGATCATTTCTTCGACTGGGGGCTGAAGGAGGAGATCGAT
amy1-1b    1 .........................................C..................
amy1-1c    1 .........................................C..A...............
amy1-1d    1 .........................................C..................
amy1-1e    1 ............................................................
amy1-1f    1 ....................................C....C..................
amy1-1g    1 .........................................C..A...............
amy1-1h    1 ...................................T.....C..T...............
amy1-1i    1 ........G................................CA.......C......T..

amy1-1a   61 CGCCTGGTGTCAATCAGGACCCGGCAGGGGATACACAGTGAGAGCAAGCTGCAAATCATA
amy1-1b   61 ............................................................
amy1-1c   61 .....A...............A.......C..............................
amy1-1d   61 ............................................................
amy1-1e   61 ............................................................
amy1-1f   61 .......................A..........................A.........
amy1-1g   61 ............................................................
amy1-1h   61 ..........................T.................................
amy1-1i   61 .....A............................................A........G

amy1-1a  121 GAGGCCGACGCCGACCTTTACCTGGCCGAGATCGATGGCAAGGTCATCGTCAAGCTCGGG
amy1-1b  121 .....T....................T........C........................
amy1-1c  121 .....T......................................................
amy1-1d  121 ............................................................
amy1-1e  121 ............................................................
amy1-1f  121 .....T.................A...........CA................A......
amy1-1g  121 ............................................................
amy1-1h  121 ....................T..C...........C........................
amy1-1i  121 .....T.................T...........C......A.................

amy1-1a  181 CCAAGATACGATGTCGGGCACCTCATTCCTCAAGGCTTCAAGGTGGTCGCGCACGGCAAT
amy1-1b  181 .............................C..............................
amy1-1c  181 ............................................................
amy1-1d  181 ............................................................
amy1-1e  181 .............................C..............................
amy1-1f  181 .............................CGG...........................A
amy1-1g  181 ............................................................
amy1-1h  181 ..............G..............GGG..............G.............
amy1-1i  181 ...C...T......G..............CGG..............C...A........A

amy1-1a  241 GACTATGCCGTATGGGAGAAAATATAAGCAAAATTATCCGAGCTGCTCCACAATTTTTCT
amy1-1b  241 .......................................A...A..............-.
amy1-1c  241 ............................................................
amy1-1d  241 ................................G...........................
amy1-1e  241 .......................................A...A..............-.
amy1-1f  241 ..T....................................A...A..............-.
amy1-1g  241 ...........................................................A
amy1-1h  241 .........................G..........C.AA...A....T..G.G....--
amy1-1i  241 .........................G..........C.AA...A....T..G.G....--

amy1-1a  301 TCCCTATA-TATGAATGTCACACCTATTAGTCCGAGCTCGTGTTGTCCACATAGTACGAT
amy1-1b  300 .T......-........C..........................................
amy1-1c  301 ........-...................................................
amy1-1d  301 ........-...................................................
amy1-1e  300 .T......-........C..........................................
amy1-1f  300 .T......-........C..........................................
amy1-1g  301 ........-.......-............-..............-...............
amy1-1h  299 ---T..C.A........A..T..A...............AC...................
amy1-1i  299 ---T..C.A........A..T..A...............AC...................

amy1-1a  360 TTTAGTACTTCCTCCATGTAAAAGTGAGGATGAGGGACATCCA
amy1-1b  359 ...........................................
amy1-1c  360 ...........................................
amy1-1d  360 ...........................................
amy1-1e  359 ...........................................
amy1-1f  359 ...........................................
amy1-1g  357 ...........................................
amy1-1h  356 ..-........................................
amy1-1i  356 ..-........................................
```

Fig. S1a Multiple sequence alignment of 462 bp *amy1-1a* – *amy1-1i* transcript sequences (cDNA). Consensus nucleotides are indicated by ‘.’

```
amy1_2a    1 GGGACCCCATGCATCTTCTACGATCATTTCTTCGACTGGGGCCTGAAGGAGGAGATAGAT
amy1_2b    1 ........G.................................A.......C.....CT..
amy1_2c    1 ............................................A...........C...
amy1_2d    1 ............................................................
amy1_2e    1 ........................................................C...
amy1_2f    1 ............................................................
amy1_2g    1 ............................................A...........C...
amy1_2h    1 ............................................A...........C...
amy1_2i    1 ............................................A...........C...
amy1_2j    1 ........G.................................A.......C.....CT..
amy1_2k    1 ..........................................A.......C.....CT..
amy1_2l    1 .........................................G..............C...
amy1_2m    1 ............................................A...........C...
amy1_2n    1 ............................................................
amy1_2o    1 ........................................................C...
amy1_2p    1 ........G.................................A.......C.....CT..
amy1_2q    1 ........G.................................A.......C.....CT..
amy1_2r    1 ........................................................C...
amy1_2s    1 ..................................G.....................C...
amy1_2t    1 ........................................A...A...........C...
amy1_2u    1 .........................................G..........G...C...
amy1_2v    1 ............................................A...........C...
amy1_2w    1 ........................................................C...

amy1_2a   61 CGCCTAGTGTCAATCAGGACCCGGCAAGGCATACACAGTGAGAGCAAGCTGCAAATCATA
amy1_2b   61 ..........................G..G....................A........G
amy1_2c   61 .....................A....G.................................
amy1_2d   61 ............................................................
amy1_2e   61 .....G.................A..G..G....................A.........
amy1_2f   61 ............................................................
amy1_2g   61 .....................A....G.................................
amy1_2h   61 .A...................A....G.................................
amy1_2i   61 .....................A....G.................................
amy1_2j   61 ..........................G..G....................A.........
amy1_2k   61 ..........................G..G................G..A........G
amy1_2l   61 .....G....................G..G....................A........G
amy1_2m   61 .....................A....G.................................
amy1_2n   61 ............................................................
amy1_2o   61 ..........T...................................G.............
amy1_2p   61 ..........................G..G....................A........G
amy1_2q   61 ......................A...G..G....................A........G
amy1_2r   61 ...................T......G.................................
amy1_2s   61 .....G.................A..G..G....................A.........
amy1_2t   61 .....................A....G.................................
amy1_2u   61 .....G....................G..G..............................
amy1_2v   61 .....................A....G.................................
amy1_2w   61 .....G....................G..G..............................

amy1_2a  121 GAGGCTGACGCCGACCTTTACCTTGCCGAGATCGATGGCAAGGTCATCGTCAAGCTCGGG
amy1_2b  121 ...................................C......A.................
amy1_2c  121 ............A.T....................C........................
amy1_2d  121 ...................................C......A.................
amy1_2e  121 .......................A...........CA................A......
amy1_2f  121 ..............................................C.............
amy1_2g  121 ............A.T....................C........................
amy1_2h  121 ............A.T....................C........................
amy1_2i  121 ............A.T....................C........................
amy1_2j  121 ............................................................
amy1_2k  121 ...................................C......A.................
amy1_2l  121 ...................................C......A.................
amy1_2m  121 ............A.T....................C........................
amy1_2n  121 ............................................................
amy1_2o  121 ............................................................
amy1_2p  121 ...................................C......A.................
amy1_2q  121 ...................................C......A.................
amy1_2r  121 T..................................C........................
amy1_2s  121 .......................A...........C........................
amy1_2t  121 ............A.T....................C........................
amy1_2u  121 .....C.................G....................................
amy1_2v  121 ............A.T.............................................
amy1_2w  121 .......................G..T........C........................

amy1_2a  181 CCAAGATATGATGTGGGGCACCTCATTCCCGGAGGCTTCAAGGTGGCCGCACACGGCAAA
amy1_2b  181 ...C...TC...................................................
amy1_2c  181 ........C.............................T.....................
amy1_2d  181 ...C...TC...................................................
amy1_2e  181 ........C.....C.......................T.....................
```

```
amy1_2f  181 ............................................................
amy1_2g  181 ...C...TC...................................................
amy1_2h  181 ........C.............................T.....................
amy1_2i  181 ........C.............................T.....................
amy1_2j  181 ............................................................
amy1_2k  181 ...C...TC...................................................
amy1_2l  181 ...C...TC...................................................
amy1_2m  181 ........C.............................T.....................
amy1_2n  181 ............................................................
amy1_2o  181 ............................................................
amy1_2p  181 ...C...TC...................G....T.....G....................
amy1_2q  181 ...C...TC...................................................
amy1_2r  181 ........C...................................................
amy1_2s  181 ........C.............................T.....................
amy1_2t  181 ........C.............................T.....................
amy1_2u  181 ........C.....C..............TCA..............T...G........T
amy1_2v  181 ............................................................
amy1_2w  181 ...C...TC...................................................

amy1_2a  241 GACTATGCCATATGGGAGAAAATATAAGAAAATTACGGGAGCAGCTCTGCAAATTAGTCA
amy1_2b  241 .........G..........................CA......................
amy1_2c  241 ........TG..........................C.........-.............
amy1_2d  241 .........G..........................CA......................
amy1_2e  241 ........TG..........................C.........-.............
amy1_2f  241 ............................................................
amy1_2g  241 .........G..........................CA......................
amy1_2h  241 ........TG..........................C.........-.............
amy1_2i  241 ........TG..................................................
amy1_2j  241 ............................................................
amy1_2k  241 .........G..........................CA......................
amy1_2l  241 .........G..........................CA......................
amy1_2m  241 ........TG..........................C.........-.............
amy1_2n  241 ....................................C.........-.............
amy1_2o  241 ............................................................
amy1_2p  241 ....G....G..........................CA....T.............A...
amy1_2q  241 .........G..................................................
amy1_2r  241 .........G..........................CA.....A................
amy1_2s  241 ........TG..........................C.........-.............
amy1_2t  241 ........TG...............................A..................
amy1_2u  241 .........G..........................CA......................
amy1_2v  241 ............................................................
amy1_2w  241 .........G..........................CA......................

amy1_2a  301 AGTTGTCCACATAGTACGATTTTAGTACTTCCTCCATGTAAAAGTGAGGATGAGGGACAT
amy1_2b  301 T...........................................................
amy1_2c  300 T...........................................................
amy1_2d  301 T...........................................................
amy1_2e  300 T...........................................................
amy1_2f  301 .........T..................................................
amy1_2g  301 T...........................................................
amy1_2h  300 T...........................................................
amy1_2i  301 ............................................................
amy1_2j  301 ............................................................
amy1_2k  301 T...........................................................
amy1_2l  301 T...........................................................
amy1_2m  300 T...........................................................
amy1_2n  300 T...........................................................
amy1_2o  301 ............................................................
amy1_2p  301 T...............TT......C...........A..G.........GA.........
amy1_2q  301 ............................................................
amy1_2r  301 T...........................................................
amy1_2s  300 T...........................................................
amy1_2t  301 ............................................................
amy1_2u  301 T...........................................................
amy1_2v  301 ............................................................
amy1_2w  301 T...........................................................

amy1_2a  361 CCA
amy1_2b  361 ...
amy1_2c  360 ...
amy1_2d  361 ...
amy1_2e  360 ...
amy1_2f  361 ...
amy1_2g  361 ...
amy1_2h  360 ...
amy1_2i  361 ...
amy1_2j  361 ...
```

```
amy1_2k  361 ...
amy1_2l  361 ...
amy1_2m  360 ...
amy1_2n  360 ...
amy1_2o  361 ...
amy1_2p  361 ...
amy1_2q  361 ...
amy1_2r  361 ...
amy1_2s  360 ...
amy1_2t  361 ...
amy1_2u  361 ...
amy1_2v  361 ...
amy1_2w  361 ...
```

Fig. S1b Multiple sequence alignment of 423 bp *amy1-2a* – *amy1-2w* transcript sequences (cDNA). Consensus nucleotides are indicated by '.'

```
amy1-3a    1 GGGACCCCATGCATCTTCTACGATCATTTCTTCGACTGGGGCCTGAAGGAGGAGATCGAT
amy1-3b    1 ........................................................A...
amy1-3c    1 ............................................A...............
amy1-3d    1 ..........................C...............................G.

amy1-3a   61 CGCCTGGTGTCAATCAGGACCCGACAGGGGATACACAGTGAGAGCAAGCTACAAATCATA
amy1-3b   61 .....A.................G..A..C....................G.........
amy1-3c   61 .....A...............A.G.....C....................G.........
amy1-3d   61 ............................................................

amy1-3a  121 GAGGCTGACGCCGACCTTTACCTAGCCGAGATCGACAGCAAGGTCATCGTCAAACTCGGG
amy1-3b  121 .......................T...........TG................G......
amy1-3c  121 ............................................................
amy1-3d  121 ............................................................

amy1-3a  181 CCAAGATACGATGTCGGGCACCTCATTCCCGGAGGCTTCAAGGTGGTCGCGCACGGCAAA
amy1-3b  181 ........T.....G...............................C...A.........
amy1-3c  181 ............................................................
amy1-3d  181 ................................G...........................

amy1-3a  241 GATTATGCCGTATGGGAGAAAATATAAGCAAAATTACCGGAGCTCATGATGTCCACGTAG
amy1-3b  241 ..C......A..........................................T.......
amy1-3c  241 ............................................................
amy1-3d  241 ............................................................

amy1-3a  301 TACGATTTAGTACTTCCTCCGTGTAAAAGTGAGGATGAGGGACATCCA
amy1-3b  301 ................................................
amy1-3c  301 ................................................
amy1-3d  301 ..............-.................................
```

Fig.S1c Multiple sequence alignment of 408 bp *amy1-3a* – *amy1-3d* transcript sequences (cDNA). Consensus nucleotides are indicated by '.'

Table S2. Isoelectric points of the partial coding 3' end *α-Amy-1* sequences calculated using Vector NTI form a total of five isoelectric point groups.

**Isoelecric Points of Partial Sequences**	**Sequences**
5.64	Amy1-1a, amy1-1b, amy1-1c, amy1-1d, amy1-1e, amy1-1g, amy1-1q
5.80	Amy1-1h
5.91	Amy1-1i, amy1-2a, amy1-2b, amy1-2d, amy1-2e, amy1-2f, amy1-2j, amy1-2l, amy1-2n, amy1-2o, amy1-2p, amy1-2t, amy1-2u, amy1-2w, amy1-3a, amy1-3b, amy1-3c
6.04	Amy1-2h
6.20	Amy1-1f, amy1-2c, amy1-2g, amy1-2i, amy1-2k, amy1-2m, amy1-2s, amy1-2v, amy1-3d

Table S3. Genbank id of five *α-Amy-1* gene sequences highly expressed during LMA in genotype SpM52.

Sequences	Genbank ID
amy1-1a	KF581187
amy1-1b	KF581188
amy1-2a	KF581189
amy1-2b	KF581190
amy1-3a	KF581191

```
amy1-1a         ATGTGGCCCTTCCCTTCCGACAAGGTCATGCAGGGATACGCCTACATCCTCACGCACCCT 60
amy1-3a         ATGTGGCCCTTCCCTTCCGACAGGGTCATGCAGGGATATGCCTACATCCTCACGCACCCA 60
amy1-2a         ATGTGGCCCTTCCCTTCCGACAGGGTCATGCAGGGATACGCCTACATCCTCACGCACCCA 60
Hvamy6-4        ATGTGGCCCTTCCCTTCTGACAGGGTCATGCAGGGATATGCCTACATCCTCACGCACCCA 60
Hvamy46         ATGTGGCCCTTCCCTTCCGACAGGGTCATGCAGGGATATGCCTACATCCTCACGCACCCA 60
Hvamy32b        ATGTGGCCATTCCCCTCCGACAAGGTCATGCAAGGCTACGCATACATCCTCACCCACCCA 60
                ******** ***** ** **** ********* ** ** ** *********** *****

amy1-1a         GGGACCCCATGCATCTTCTACGATCATTTCTTCGACTGGGGGCTGAAGGAGGAGATCGAT 120
amy1-3a         GGGACCCCATGCATCTTCTACGATCATTTCTTCGACTGGGGCCTGAAGGAGGAGATCGAT 120
amy1-2a         GGGACCCCATGCATCTTCTACGATCATTTCTTCGACTGGGGCCTGAAGGAGGAGATAGAT 120
Hvamy6-4        GGGACGCCATGCATCTTCTACGATCATTTCTTCGACTGGGGCCTGAAGGAGGAGATCGAT 120
Hvamy46         GGGACCCCATGCATCTTCTACGATCATTTCTTCGACTGGGGCTTGAAGGAGGAGATCGAT 120
Hvamy32b        GGCACCCCATGCATCTTCTACGACCATTTCTTTAACTGGGGGTTCAAGGATGAGATCGCG 120
                ** ** ***************** ********  *******  * ***** ***** *

amy1-1a         CGCCTGGTGTCAATCAGGACCCGGCAGGGGATACACAGTGAG-AGCAAGCTGCAAATCAT 179
amy1-3a         CGCCTGGTGTCAATCAGGACCCGACAGGGGATACACAGTGAG-AGCAAGCTACAAATCAT 179
amy1-2a         CGCCTAGTGTCAATCAGGACCCGGCAAGGCATACACAGTGAG-AGCAAGCTGCAAATCAT 179
Hvamy6-4        CGCCTGGTGTCAGTCAGGACCCGGCACGGGATACACAACGAG-AGCAAGCTGCAAATCAT 179
Hvamy46         CGTCTGGTGTCAATCAGGACCCGACAGGGGATACACAGTGAG-AGCAAGCTGCAGATCAT 179
Hvamy32b        GCGCTGGTGGCGATCAGGAAGCGAAACGGCAT-CACGGCGACGAGTGCGCTCGAGATCCT 179
                   ** *** *  ******  **  * ** ** ***   **  **   ***  * *** *

amy1-1a         AGAGGCCGACGCCGACCTTTACCTGGCCGAGATCGATGGCAAGGTCATCGTCAAGCTCGG 239
amy1-3a         AGAGGCTGACGCCGACCTTTACCTAGCCGAGATCGACAGCAAGGTCATCGTCAAACTCGG 239
amy1-2a         AGAGGCTGACGCCGACCTTTACCTTGCCGAGATCGATGGCAAGGTCATCGTCAAGCTCGG 239
Hvamy6-4        AGAGGCCGACGCCGACCTTTATCTCGCCGAGATCGACGGCAAGGTCATCGTCAAGCTCGG 239
Hvamy46         GGAGGCCGACGCCGACCTTTACCTTGCCGAGATCGACGGCAAGGTCATCGTCAAGCTCGG 239
Hvamy32b        CATGCACGAAGGAGACGCCTACGTCGCGGAGATCGACGGCAAGGTGGTGGTGAAAATCGG 239
                   *   ** *  ***   **  * ** ********  *******  * ** **  ****

amy1-1a         GCCAAGATACGATGTCGGGCACCTCATTCCTCAAGGCTTCAAGGTGGTCGCGCACGGCAA 299
amy1-3a         GCCAAGATACGATGTCGGGCACCTCATTCCCGGAGGCTTCAAGGTGGTCGCGCACGGCAA 299
amy1-2a         GCCAAGATATGATGTGGGGCACCTCATTCCCGGAGGCTTCAAGGTGGCCGCACACGGCAA 299
Hvamy6-4        GCCAAGATACGATGTGGGGAACCTCATTCCGGGAGGCTTCAAGGTGGCCGCGCACGGCAA 299
Hvamy46         GCCAAGATACGATGTCGGACACCTCATTCCTGAAGGCTTCAAGGTGGTCGCGCATGGCAA 299
Hvamy32b        GACCAGGTACGACGTCGGGGCGGTGATCCCGGCTGGGTTCGCAACGTCGGCGCACGGCAA 299
                * * ** ** ** ** **     * ** **    ** ***     *   ** ** *****

amy1-1a         TGACTAT-GCCGTATGGGAGAAAATA-------TAAGCAAAATTATCCGAGCTG----CT 347
amy1-3a         AGATTAT-GCCGTATGGGAGAAAATA-------TAAGCAAAATTA-CCG----------- 339
amy1-2a         AGACTAT-GCCATATGGGAGAAAATA-------TAAG-AAAATTACGGGAGCAG----CT 346
Hvamy6-4        TGACTAT-GCCGTATGGGAGAAAATA-------TGAGCAAAATTGCGAGAGCAG----CT 347
Hvamy46         TGACTAT-GCCGTATGGGAGAAAGTAT------AAAGCAAAATTAACGGAGCGG----CT 348
Hvamy32b        GGACTAACGCCGTATCGGAGAAGCAGCTGCCGCGGCGACACTACAACGGAGCTGAAGCCT 359
                 ** **  *** *** ******              *  *        *

amy1-1a         CCACAATTTTTCTTCCCTATATATGAATGTCACACCTATTAGTCCGAGCTCGTGTTGTCC 407
amy1-3a         ---------------------------------------------GAGCTCATGATGTCC 354
amy1-2a         CTGCAAAT--------------------------------------TAGTCAAGTTGTCC 368
Hvamy6-4        CTACAAGTTCCTATATG--------------ATACATATTAGTCCGAGCTCACGCTGTTC 393
Hvamy46         CTACAAATT-------------------------------AGTCCGAGCTCGTGTTGTCC 377
Hvamy32b        CCACTCATCCACCATTC--------------------AATCGAGCATGCATGAATTTTCC 399
                                                                       * * *

amy1-1a         ACA----TAGTACGATTTTAGTACTTCCTC-CATGTAAAAGTG-AGGATGAGG-----GA 456
amy1-3a         ACG----TAGTACGATTT-AGTACTTCCTC-CGTGTAAAAGTG-AGGATGAGG-----GA 402
amy1-2a         ACA----TAGTACGATTTTAGTACTTCCTC-CATGTAAAAGTG-AGGATGAGG-----GA 417
Hvamy6-4        ACA----TAGTACAATTT-AATACTTCCTC-CATGTAAAAGTG-AGGATGAGG-----GA 441
Hvamy46         ACA----TAGTACGATTTTAGTACTTCCTC-CATGTAAAAAAGGAGGATGAGG-----GA 427
Hvamy32b        AAAATAATGATTCACTTCTGCTATAGACACGCAGATATATATGATTAATTACGTAGTATA 459
                *      *  * *  **    **    * * *   ** *   *    ** * *      *

amy1-1a         CATCCA 462
amy1-3a         CATCCA 408
amy1-2a         CATCCA 423
Hvamy6-4        CATGCA 447
Hvamy46         CATCCA 433
Hvamy32b        CTTCTG 465
                * *
```

Fig S2. Multiple sequence alignment of the partial sequence of wheat high pI α-amylase with barley high pI α-amylase partial sequence (*Hvamy6-4* and *Hvamy46*)

and barley low pI α-amylase partial sequence (*Hvamy32b*). Consensus nucleotides are indicated by asterices (*).

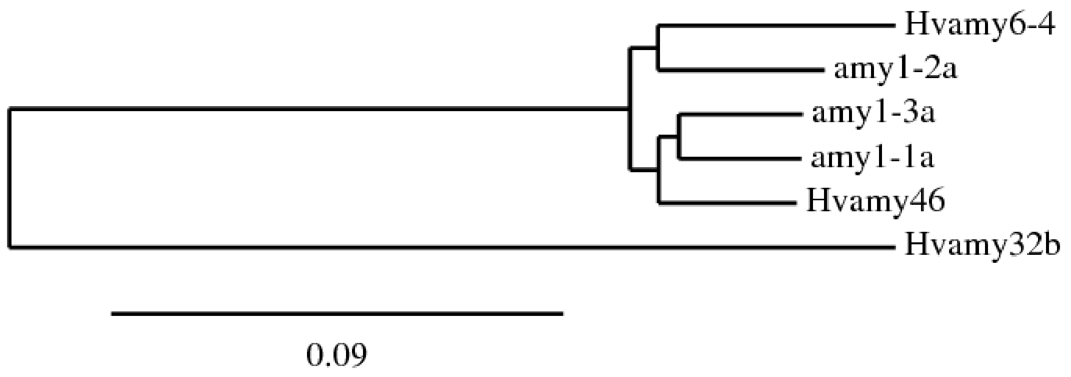

Fig. S3 Phylogenetic tree of the high pI α-amylase genes of wheat (*amy1-1*, *amy1-2* and *amy1-3*) and barley (*Hvamy46* and *Hvamy6-4*), and the barley low pI α-amylase gene *Hvamy32*. Phylogenetic tree generated using online program TreeDyn 198.3.

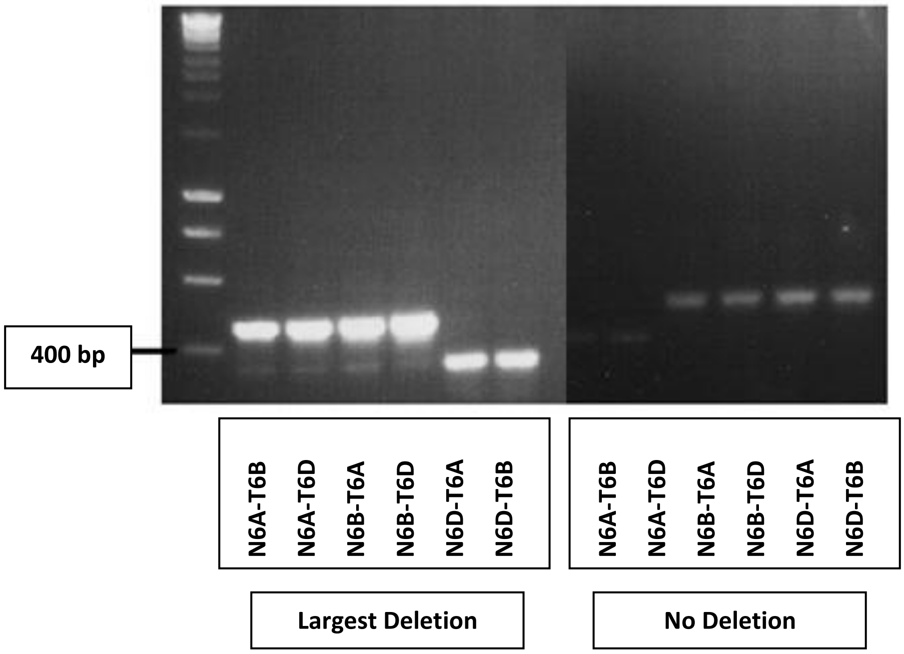

Fig. S4 Screening of the group 6 nullisomic-tetrasomic lines of Chinese Spring for gene family members with the largest region of deletion and the region with no deletion.
